# Plethysmographic Loops: A Window on the Lung Pathophysiology of COPD Patients

**DOI:** 10.3389/fphys.2018.00484

**Published:** 2018-05-01

**Authors:** Dejan Radovanovic, Matteo Pecchiari, Fabio Pirracchio, Camilla Zilianti, Edgardo D’Angelo, Pierachille Santus

**Affiliations:** ^1^Dipartimento di Scienze Biomediche e Cliniche Luigi Sacco, Università degli Studi di Milano, Milan, Italy; ^2^Dipartimento di Fisiopatologia Medico-Chirurgica e dei Trapianti, Università degli Studi di Milano, Milan, Italy

**Keywords:** plethysmographic loops, airway resistance, chronic obstructive pulmonary disease, respiratory function tests, respiratory pathophysiology

## Abstract

Plethysmographic alveolar pressure-flow (*P*_alv_–*F*) loops contain potentially relevant information about the pathophysiology of chronic obstructive pulmonary disease (COPD), but no quantitative analysis of these loops during spontaneous breathing has ever been performed. The area of the loop’s inspiratory (*A*_ins_) and expiratory portion (*A*_exp_), and the difference between the end-expiratory and end-inspiratory alveolar pressure (Δ*P*_alv_) were measured in 20 young, 20 elderly healthy subjects, and 130 stable COPD patients. *A*_ins_ and Δ*P*_alv_ increased by 55 and 78% from young to elderly subjects, and by 107 and 122% from elderly subjects to COPD patients, reflecting changes in mechanical heterogeneity, lung-units recruitment/derecruitment, and possibly air trapping occurring with aging and/or obstructive disease. *A*_exp_ increased by 38% from young to elderly subjects, and by 198% from elderly subjects to COPD patients, consistent with the additional contribution of tidal expiratory flow-limitation, which occurs only in COPD patients and affects *A*_exp_ only. In COPD patients, *A*_exp_ and Δ*P*_alv_ showed a significant negative correlation with VC, FEV_1_, IC, and a significant positive correlation with RV/TLC. The results suggest that the analysis of plethysmographic *P*_alv_–F loops provides an insight of the pathophysiological factors, especially tidal expiratory flow-limitation, that affect lung function in COPD patients.

## Introduction

**FIGURE 1:**
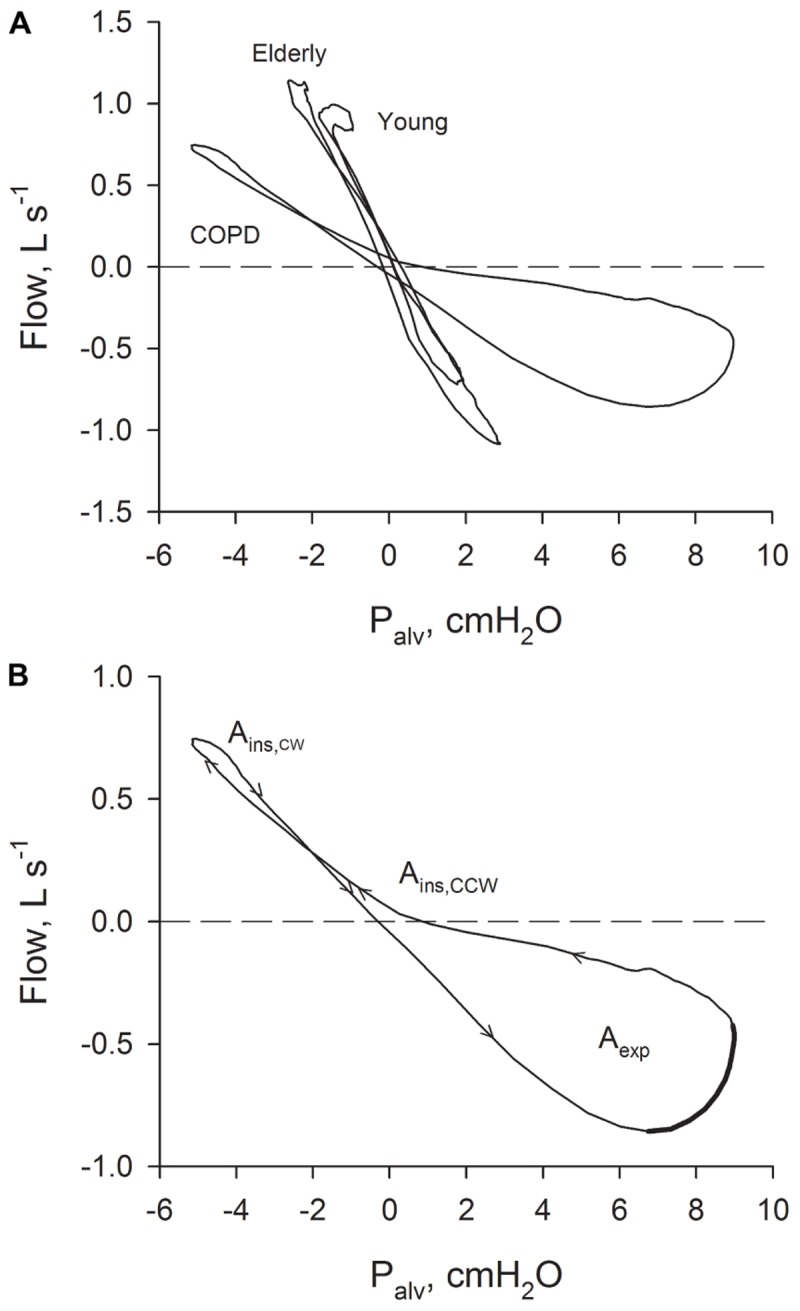
**(A)** Relations between *P*_alv_–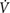 recorded in a young healthy subject, in an old healthy subject and in a patient with severe COPD. **(B)** Relation between *P*_alv_–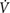 recorded in a patient with severe COPD with the indication of the sense of rotation (arrows). *A*_ins_: inspiratory area; *A*_exp_: expiratory area. The part of the expiratory *P*_alv_–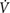 suggestive of the presence of expiratory flow-limitation (where flow is decreasing while driving pressure is increasing) is indicated with a thicker line.

In 1955 Arthur DuBois devised a method for the assessment of airway resistance during panting based on the measurement of flow (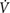) with a flowmeter and on the estimation of alveolar pressure (*P*_alv_) from the pressure change inside a constant volume plethysmograph (*P*_box_); it was immediately noted that the *P*_alv_–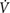 relation is almost a line in normal subjects, but becomes a loop in some patients with chronic obstructive pulmonary disease (COPD) or asthma (DuBois et al., 1956) (examples of *P*_alv_–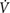 relations recorded with a modern plethysmograph in two healthy subjects and a COPD patient are shown in **Figure 1**). In fact, studies that investigated the relationship between 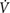 and the non-elastic component of transpulmonary pressure (*P*_L,dyn_) found that, in contrast to normal subjects, the relation between *P*_L,dyn_ and 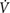 in emphysematous patients manifested an expiratory looping at rest (Mead and Whittenberger, 1953; Mead et al., 1955).

In modern plethysmographs, *P*_box_ is usually displayed as shift volume (Δ*V*_S_), that is the change in lung volume due only to compression or decompression of gas inside of the lung. Since plethysmographic measurements of airway resistance entered the clinical practice, looping of the Δ*V*_S_–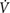 diagram has become familiar to respiratory physicians, who relate this finding to the presence of obstruction (Criée et al., 2011). However, a number of factors can be responsible for the appearance and rotational direction of *P*_alv_–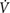 loops. In expiration, mechanical heterogeneity, air trapping, recruitment/derecruitment of lung units, and expiratory flow-limitation produce a counterclockwise (CCW) loop, whereas in inspiration, mechanical heterogeneity and air trapping produce a CCW loop, while recruitment/derecruitment causes a clockwise (CW) loop (Jaeger and Bouhuys, 1969; Matthys, 1972). Mechanical heterogeneity refers to an uneven distribution of the mechanical properties in the different lung regions. Firstly described in terms of pure viscous resistances and compliances (Otis et al., 1956), heterogeneity of the viscoelastic and plastoelastic characteristics of lung tissue should also be involved (Suki and Bates, 2011).

To date a quantitative analysis of the *P*_alv_–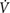 loops during spontaneous breathing at rest has never been performed. Presumably, this occurred because most of the commercially available plethysmographs measure *P*_alv_ during panting, an unnatural modality of breathing, while the BTPS rebreathing technique is not used in the clinical settings due to hygienic concerns. The availability of plethysmographs that measure Δ*V*_S_ continuously during spontaneous breathing raises the possibility to exploit the *P*_alv_–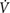 loops to further characterize the mechanical alterations of the respiratory system.

We hypothesize that, if the *P*_alv_–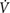 loops provided by the plethysmograph are free from major artifacts, in expiration the loop should rotate CCW, both in healthy and COPD subjects, as in expiration all the factors potentially responsible for the genesis of the loop induce a CCW rotation. In contrast, in inspiration loops may rotate CW or CCW, because in inspiration the loop-generating factors have opposite effects on the sense of rotation. For the same reasons, we expect the expiratory loop to be bigger than the inspiratory one, especially in COPD patients who may show tidal expiratory flow-limitation at rest, a loop generating factor present only in expiration.

The aims of the present study therefore were: (A) to characterize numerically the *P*_alv_–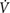 loops obtained during spontaneous breathing in healthy subjects and in COPD patients; (B) to assess if the recorded loops are compatible with what is currently known about the pathophysiological phenomena responsible for the looping; and (C) to investigate the relationship between loops’ parameters and routine lung function testing.

## Materials and Methods

### Experimental Subjects

The data for this observational study were collected from 20 young and 20 elderly healthy subjects enrolled in a physiological study (Pecchiari et al., 2016), and from 130 COPD patients who participated to a prospective, observational, multicenter study (Santus et al., 2016). Both studies were conducted according to the amended Declaration of Helsinki and approved by the local ethical committee (Fondazione Salvatore Maugeri–654 CEC e 717 CEC). All participants gave written, informed consent. COPD patients were allowed to take their usual inhaled therapy.

For COPD patients, inclusion criteria were: (a) a confirmed diagnosis of COPD, (b) stable clinical conditions, and (c) the ability to perform pulmonary function tests. Exclusion criteria were (a) impaired cognitive function (Mini-Mental State Examination score <26), (b) a current diagnosis of neoplastic or musculoskeletal diseases, or previous lobectomy, (c) a mixed obstructive-restrictive ventilatory pattern, (d) history of asthma, (e) a confirmed diagnosis of obstructive sleep apnoea, (f) a BMI > 34 and (g) recent cardiothoracic surgery or NYHA III or IV functional class heart failure.

### Measurements

Lung function tests were performed according to the American Thoracic Society/European Respiratory Society (ATS/ERS) guidelines (Wanger et al., 2005).

Static and dynamic lung volumes, and airway resistance were measured with a constant-volume plethysmograph (MasterScreen Body Plethysmograph, Erich Jaeger GmbH, Würzburg, Germany). Intrathoracic gas volume (ITGV) was measured close to the end-expiratory lung volume during quiet breathing.

In COPD patients, oxygen (PaO_2_) and carbon dioxide pressure (PaCO_2_) were measured on blood samples taken from the radial artery while patients were comfortably seated, breathing quietly room air, after resting for at least 15 min (GEM Premier 3000; Instrumentation Laboratory, Lexington, MA, United States). Exercise tolerance and dyspnea were evaluated using the 6 min walk test (6MWT) and the Borg dyspnea scale (BDS), according to ATS recommendations (ATS Committee on Proficiency Standards for Clinical Pulmonary Function Laboratories, 2002).

### Data Analysis

During the assessment of airway resistance, the plethysmographic Δ*V*_S_ and 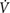, sampled at 50 Hz for 10 consecutive breaths, were stored as ASCII files. These files were retrieved for each patient and analyzed with a custom-built LabView program (National Instruments, Austin, TX, United States), performing the following operation:

(a) Conversion of Δ*V*_S_ into *P*_alv_ for all breaths, according to the following equation:

$$P_{alv,t}\;=\;-\frac{P_{B}\Delta V_{S,t}}{V_{rs,t}+\Delta V_{S,t}}$$

where *P*_alv_ is alveolar pressure minus *P*_B_, *P*_B_ barometric minus vapor pressure, Δ*V*_S_ the shift volume, and *V*_rs_ the volume of the respiratory system, calculated as the sum of the ITGV and the time integral of the flow;

(b) Discard of abnormal breaths (cough or sigh) by an operator blind to the identity of the subject;

(c) Averaging of the acquired inspirations and expirations after normalization with respect to their duration to obtain the subject’s representative breath.

Subsequently, the following parameters were assessed:

(a) Tidal volume (*V*_T_), duration of inspiration (*T*_I_) and of expiration (*T*_E_),

(b) Overall sense of rotation (CW or CCW), calculated by the continuous numerical integration of *P*_alv_ on 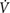 for the whole inspiration, and, separately, for the whole expiration. In this way, if the integral is negative, the overall sense of rotation is CW, if the integral is positive, the overall sense of rotation is CCW;

(c) Area of the inspiratory (*A*_ins_) and of the expiratory (*A*_exp_) loop (the inspiratory loop could have a part rotating CCW and a part rotating CW: in this case *A*_ins_ was obtained as the sum of the two areas);

(d) Difference between the alveolar pressures at the beginning and at the end of the inspiration (Δ*P*_alv_). The beginning and the end of the inspiration were defined in terms of zero 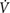.

(e) Total (*R*_tot_), inspiratory (*R*_ins_) and expiratory (*R*_esp_) airway resistance (Ulmer and Reif, 1965).

### Statistics

Data, presented as mean ± SD or as median (IQR), were analyzed using SPSS 23 (SPSS Inc., Chicago, United States). Normality of distributions was assessed by Kolmogorov–Smirnov or Shapiro–Wilk test. Differences between groups were investigated with analysis of variance (ANOVA) or by Kruskal–Wallis test, followed by *post hoc* analysis. Relationships between variables were assessed by means of linear regression analysis or Spearman’s rank correlation. In all instances, statistical significance was taken at *p* ≤ 0.05.

## Results

The anthropometric characteristics together with spirometric and plethysmographic parameters of healthy subjects and COPD patients are shown in **Table 1**. According to the GOLD classification of airflow severity in COPD (Global Initiative for Chronic Obstructive Pulmonary Disease (GOLD), 2018) 12 patients were GOLD 1 (9%), 50 GOLD 2 (39%), 48 GOLD 3 (37%), and 20 GOLD 4 (15%).

**Table 1:** Anthropometric characteristics, spirometric and plethysmographic parameters of healthy subjects and COPD patients.

	Young	Elderly	COPD
M / F	14/6	17/3	90/40
Age, yrs	23 ± 3	71 ± 4	72 ± 7
Height, m	1.76 ± 0.10	1.75 ± 0.08	1.65 ± 0.09
Weight, kg	73 ± 16	79 ± 12	72 ± 17
BMI	23 ± 4	26 ± 2	26 ± 5
Former/active smokers	0/0	0/0	128/2
FEV_1_, L %pred	4.44 ± 0.86 105 ± 11	2.89 ± 0.58 100 ± 16	1.19 ± 0.52^∗∗^ 51 ± 20^∗∗^
FVC, L %pred	5.28 ± 1.09 106 ± 11	3.85 ± 0.69 103 ± 12	2.19 ± 0.69^∗∗^ 73 ± 19^∗^
FEV_1_/ FVC, %	85 ± 7	76 ± 11	54 ± 13^∗∗^
IC, L %pred	3.41 ± 0.84 107 ± 23	3.00 ± 0.56 101 ± 13	2.15 ± 0.73^∗∗^ 86 ± 23
VC, L %pred	5.31 ± 1.08 103 ± 11	4.10 ± 0.78 105 ± 11	2.67 ± 0.80^∗∗^ 85 ± 19^∗∗^
TLC, L %pred	6.83 ± 1.19 103 ± 10	6.81 ± 1.01 102 ± 12	6.51 ± 1.44 112 ± 18
RV, L %pred	1.45 ± 0.30 94 ± 18	2.67 ± 0.55 104 ± 20	3.85 ± 1.15^∗∗^ 162 ± 45^∗∗^
ITGV, L %pred	3.49 ± 0.85 102 ± 20	3.70 ± 0.90 99 ± 23	4.36 ± 1.27 133 ± 33^∗^
*R*_tot_, cmH_2_O.L^-1^.s %pred	2.1 ± 0.6 69 ± 20	2.3 ± 0.7 80 ± 27	6.9 ± 3.7^∗∗^ 227 ± 121^∗∗^
*R*_ins_, cmH_2_O.L^-1^.s	1.8 ± 0.5	2.2 ± 0.7	5.7 ± 2.9^∗∗^
*R*_exp_, cmH_2_O.L^-1^.s	2.6 ± 0.7	3.1 ± 1.1	13.0 ± 11.8^∗∗^

*Values are mean ± SD. FEV_1_: forced expiratory volume in 1 s; FVC: forced vital capacity; IC: inspiratory capacity; VC: slow vital capacity; TLC: total lung capacity; RV: residual volume; ITGV: intrathoracic gas volume; R_tot_: airway resistance; R_ins_: inspiratory airway resistance; R_exp_: expiratory airway resistance. Lung function parameters were compared between COPD patients and elderly healthy controls: ^∗^p < 0.05, ^∗∗^p < 0.001.*

Compared to elderly subjects, COPD patients showed significantly worse lung function parameters, except for total lung capacity, indicating the presence of obstruction (FEV_1_ 51 ± 20 vs. 100 ± 16%, *P* < 0.001, and *R*_tot_ 6.9 ± 3.7 vs. 2.3 ± 0.7 cmH_2_O.L^-1^.s), and hyperinflation and gas trapping (FRC 133 ± 33 vs. 99 ± 23%, *P* < 0.001, and RV 162 ± 45 vs. 104 ± 20%, *P* < 0.001). Patients were hypoxemic (PaO_2_ 72 ± 9 mmHg, SaO_2_ 94 ± 2%) and normocapnic (PaCO_2_ 42.6 ± 6.8 mmHg), and the majority had a reduced exercise tolerance (6MWT 344 ± 116 m, 75 ± 24%pred). Dyspnea sensation was elevated immediately after exercise [BDS 3.0 (3.3)].

**FIGURE 2:**
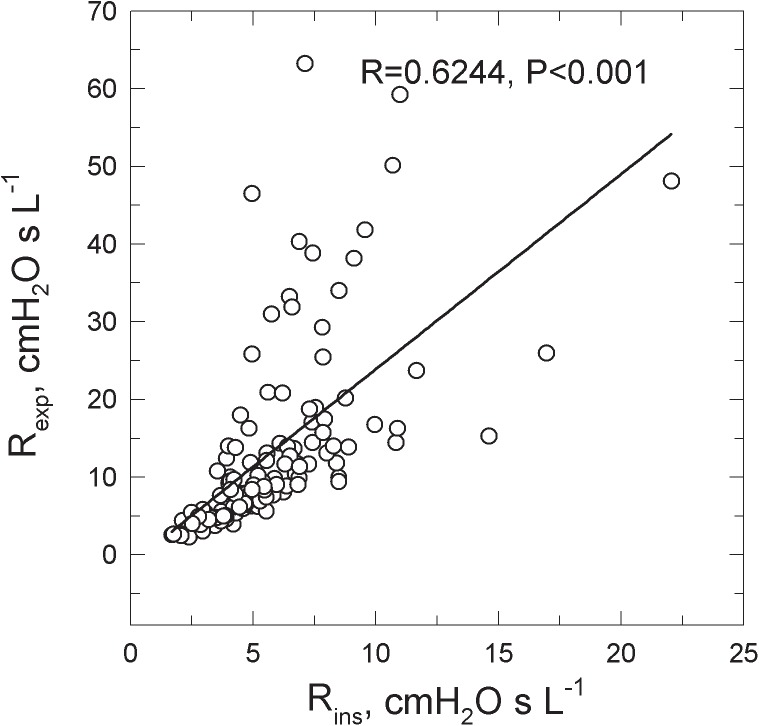
Relations between the inspiratory (*R*_ins_) and the expiratory (*R*_exp_) resistance measured in 130 COPD patients spontaneously breathing at rest.

*R*_exp_ was greater than *R*_ins_ in all groups (**Table 1**). **Figure 2** shows the *R*_exp_–*R*_ins_ relation obtained in COPD patients.

While no difference in breathing pattern occurred between healthy young and elderly subjects, *T*_I_ was shorter and the mean inspiratory flow was greater in COPD patients than in healthy controls (**Table 2**).

**Table 2:** Breathing pattern parameters in healthy and COPD subjects.

	Young	Elderly	COPD
*V*_T_, L	0.75 ± 0.21	0.72 ± 0.14	0.76 ± 0.26
*T*_I_, s	1.45 ± 0.23	1.50 ± 0.31	1.29 ± 0. 39^†^
*T*_E_, s	2.01 ± 0.36	2.15 ± 0.73	2.08 ± 0.77
RR, min^-1^	18 ± 3	18 ± 5	19 ± 6
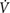 _E_, L.min^-1^	12.9 ± 2.7	12.7 ± 4.3	14.1 ± 4.3
mF_I_, L.s^-1^	0.51 ± 0.10	0.50 ± 0.12	0.61 ± 0.17^∗^
mF_E_, L.s^-1^	0.37 ± 0.08	0.38 ± 0.16	0.39 ± 0.13

*Values are mean ± SD. V_T_: tidal volume; T_I_: inspiratory duration; T_E_: expiratory duration; 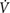
_E_: pulmonary ventilation; RR: respiratory rate; mF_I_: mean inspiratory flow; mF_E_: mean expiratory flow. Breathing pattern parameters of young and elderly healthy subjects were similar. COPD patients vs. elderly subjects: ^†^P = 0.057; ^∗^P = 0.010.*

### *P*_alv_ – 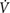 Loop-Derived Parameters

The results of the analysis performed on the average breaths are reported in **Table 3**. **Figure 1A** shows typical *P*_alv_–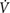 plots recorded in a young healthy subject, an elderly healthy subject, and a severe COPD patient, the sense of rotation being indicated by the arrows in **Figure 1B**. The inspiratory loop had a complex shape, and its overall sense of rotation was variable, being CCW in about half of both healthy subjects and COPD patients. In contrast, the expiratory loop ran CCW in all healthy subjects and in all but one COPD patients, in whom the expiratory loop was exceptionally small (*A*_exp_ = 0.056 cmH_2_O L s^-1^).

**Table 3:** Loop-derived parameters in healthy and COPD subjects.

	Young	Elderly	COPD
*A*_ins_, cmH_2_O.L.s^-1^	0.121 (0.076)^†^	0.188 (0.151)^†^	0.388 (0.345)^†∗^
*A*_exp_, cmH_2_O.L.s^-1^	0.313 (0.174)	0.432 (0.274)	1.287 (1.778)^∗^
Δ*P*_alv_, cmH_2_O	0.18 (0.21)	0.32 (0.14)	0.71 (0.83) ^∗^

*Values are median (IQR). A_ins_: area of the inspiratory loop; A_exp_: area of the expiratory loop; ΔP_alv_: difference between end-expiratory and end-inspiratory alveolar pressure. All values were significantly different from zero. ^†^P < 0.01 compared to corresponding A_exp_ values; ^∗^P ≤ 0.01 compared to young and elderly healthy subjects.*

No significant difference was detected in terms of loop-derived parameters between young or elderly healthy subjects. Both *A*_ins_ and *A*_exp_ were much greater in COPD patients than in healthy subjects, while *A*_exp_ was greater than *A*_ins_ in all groups, (**Table 3**).

In all groups, *P*_alv,ei_ and *P*_alv,ee_ were significantly lower and greater than zero, respectively. Their difference (Δ*P*_alv_) was significantly larger in COPD patients than in normal subjects, and larger in elderly than young healthy subjects, though not significantly (**Table 3**). Δ*P*_alv_ correlated with both *A*_ins_ (*R*_S_ = 0.714, *P* < 0.001) and *A*_exp_ (*R*_S_ = 0.716, *P* < 0.001). Both *A*_exp_ and Δ*P*_alv_ were greater in GOLD 4 patients than in the other subgroups (*P* = 0.008 and 0.022, respectively).

### Loop-Derived Parameters and Variables of Lung Function Tests

The correlations between the loop-derived parameters and the parameters from spirometry, plethysmography, 6MWT, and arterial blood gases obtained in COPD patients are shown in **Table 4**.

**Table 4:** Dependencies of loop-derived parameters in COPD patients.

	*A*_ins_ cmH_2_O⋅L⋅s^-1^		*A*_exp_ cmH_2_O⋅L⋅s^-1^		Δ*P*_alv_ cmH_2_O	
	R_S_	P	R_S_	P	R_S_	P
*R*_ins_, cmH_2_O.L^-1^.s	**0.414**	**<0.001**	**0.493**	**<0.001**	**0.578**	**<0.001**
*R*_exp_, cmH_2_O.L^-1^.s	**0.405**	**<0.001**	**0.627**	**<0.001**	**0.617**	**<0.001**
FEV_1_, L	**-0.177**	**0.044**	**-0.341**	**<0.001**	**-0.347**	**<0.001**
FVC, L	**-0.230**	**0.008**	**-0.360**	**<0.001**	**-0.427**	**<0.001**
FEV_1_/FVC, %	-0.057	0.518	-0.143	0.105	-0.084	0.343
IC, L	-0.084	0.339	**-0.194**	**0.027**	**-0.244**	**0.005**
VC, L	**-0.205**	**0.019**	**-0.279**	**0.001**	**-0.403**	**<0.001**
TLC, L	-0.045	0.608	-0.041	0.640	-0.137	0.119
ITGV, L	-0.024	0.787	0.065	0.465	0.064	0.469
RV, L	0.053	0.549	0.145	0.100	-0.049	0.580
RV/TLC, %	**0.175**	**0.047**	**0.321**	**<0.001**	**0.301**	**<0.001**
Pa_CO2_, mmHg	0.022	0.800	0.021	0.814	**0.174**	**0.048**
Pa_O2_, mmHg	-0.040	0.650	-0.069	0.432	-0.073	0.408
6MWT, m	**-0.193**	**0.028**	**-0.245**	**0.005**	**-0.285**	**0.001**
Borg Score	0.138	0.117	**0.202**	**0.021**	0.117	0.185

*A_ins_: area of the inspiratory loop; A_exp_: area of the expiratory loop; ΔP_alv_: difference between end-expiratory and end-inspiratory alveolar pressure; FEV_1_: forced expiratory volume in 1 s; FVC: forced vital capacity; IC: inspiratory capacity; VC: slow vital capacity; TLC: total lung capacity; ITGV: intrathoracic gas volume; RV: residual volume; V_A_: alveolar volume by He dilution method; DL_CO_: diffusion capacity for carbon monoxide; K_CO_: transfer coefficient for carbon monoxide; 6MWT: 6 min walking test. Bold numbers indicate significant correlations.*

Both *A*_ins_ and *A*_exp_ were negatively correlated with FVC, VC, FEV_1_, and 6MWT, positively correlated with RV/TLC%, but unrelated to arterial blood gases. Additionally *A*_exp_ was negatively correlated with IC, and positively correlated with Borg score.

Δ*P*_alv_ correlated with static and dynamic lung volumes as *A*_exp_ did.

*A*_ins_, *A*_exp_ and Δ*P*_alv_ were positively correlated with both *R*_ins_ and *R*_exp_ (**Table 4**).

## Discussion

This is the first study that has quantitatively analyzed the *P*_alv_–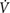 loops recorded in healthy subjects and COPD patients breathing spontaneously at rest with a commercially available constant volume-plethysmograph. It has shown that although the area of the expiratory portion of *P*_alv_–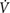 loops exceeds that of the inspiratory portion both in normal subjects and COPD patients, this discrepancy is markedly enhanced in COPD patients, indicating that among the several pathophysiological factors which can produce such loops, tidal expiratory flow limitation appears to be the most effective, as it affects expiration only and is often present in COPD patients but not in normal subjects (Pecchiari et al., 2016).

Previous investigations were focused on the qualitative study of the shape and magnitude of the *P*_alv_–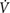 loop in relation to the underlying respiratory disease (Islam and Ulmer, 1971; Matthys, 1972), and only airway resistance (Kostianev and Ivanova, 1993), or the phase shift between *P*_alv_ and 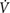 (Huckauf and Hüttemann, 1972) were numerically characterized. Notably, some of these studies were performed during rebreathing of gas mixtures in BTPS conditions, a dismissed procedure in clinical practice. More recently, an accurate characterization of the specific resistance loops has been carried out in healthy subjects, asthmatic and COPD patients by Topalovic et al. (2017). The analysis involved only the expiratory part of the loops, and parameters were extracted to describe their shape. Furthermore, the experiments were conducted with the subjects breathing at a fixed respiratory rate of 1 Hz, a condition which, relative to normal, quite breathing, can produce important changes in lung mechanics, especially in COPD patients (Loring et al., 2009). These methodological differences prevent the comparison between the present and Topalovic et al. (2017) results.

In healthy young subjects during spontaneous breathing at rest, mechanical heterogeneity should be the only source of *P*_alv_–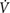 looping, because recruitment/derecruitment and tidal expiratory flow limitation are absent (Pecchiari et al., 2016), and there is no gas trapping. Simulations of *P*_alv_–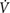 loops in the erect subject using a distribution of regional time constants compatible with available distribution of regional lung volumes (Milic-Emili et al., 1966), quasi-static transpulmonary pressure (Pecchiari et al., 2016) and airway conductance–lung volume relationships (D’Angelo et al., 2000) suggest that both *A*_exp_ and *A*_ins_ should be small, and smaller in young than elderly subjects, in line with data presented in **Table 3**. Indeed, aging causes mechanical heterogeneity to increase, as both phase III slope of the single breath nitrogen test and difference between static and dynamic compliance are increased substantially in elderly healthy subjects (Buist and Ross, 1973; Begin et al., 1975). While linear models produce loops of the same inspiratory and expiratory areas, *A*_ins_ was significantly smaller than *A*_exp_ both in young and elderly subjects (**Table 3**). This can be related to the significantly lower inspiratory than expiratory airway resistance both in young and elderly subjects (**Table 1**). Additionally, recruitment/derecruitment of pulmonary units takes place with aging, though in a minority (∼25%) of subjects (Pecchiari et al., 2016), while gas trapping might have also occurred, at least in more elderly subjects. This could explain the greater increase of *A*_exp_ than *A*_ins_ in the elderly relative to young subjects (**Table 3**). Indeed, the sense of rotation imparted to the *P*_alv_–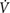 loop by recruitment/derecruitment on the one hand, and mechanical heterogeneity or gas trapping on the other hand are opposite only during inspiration (Jaeger and Bouhuys, 1969; Matthys, 1972), thus subtracting to *A*_ins_.

Chronic obstructive pulmonary disease patients exhibited substantially enlarged *P*_alv_–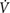 loops (**Figure 1** and **Table 3**). Mechanical heterogeneity should be in fact enhanced in this disease, as well as the presence in the *V*_T_ range of recruitment/derecruitment, expiratory flow limitation, and possibly gas trapping (Eltayara et al., 1996; D’Angelo et al., 2000; Milite et al., 2009; Pecchiari et al., 2016, 2017). A connection between *A*_exp_ and gas trapping could be in fact suggested by the significant negative correlation of *A*_exp_ with FVC, VC, and IC and the significant positive correlation with RV/TLC (**Table 4**). The sense of rotation imparted by these factors to the loops (Jaeger and Bouhuys, 1969; Matthys, 1972), and the fact that tidal expiratory flow limitation contributes only to the expiratory loop, explain both the enlargement of the *P*_alv_–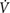 loops and the substantially greater increase of *A*_exp_ than *A*_ins_ (**Table 3**). Moreover, the marked deformation of the expiratory loop and its direction (**Figure 1**), together with the fact that the other factors have the potential to originate undistorted loops, suggest that expiratory flow limitation should be the major contributor to the formation of the expiratory part of the loops. This suggestion is also supported by the significant negative correlation between *A*_exp_ and IC or FEV_1_ (**Table 4**), because dynamic hyperinflation is more frequently observed in the presence of tidal expiratory flow limitation, and FEV_1_ is lower in expiratory flow-limited than non flow-limited COPD patients (Eltayara et al., 1996; D’Angelo et al., 2009). It is important to note that the breathing pattern did not differ between healthy subjects and COPD patients (**Table 2**), thus excluding a possible confounding factor.

*P*_alv,ei_ and *P*_alv,ee_ should represent the average of the volume-weighted alveolar pressures existing in the different pulmonary units at the inspiratory-to-expiratory and expiratory-to-inspiratory transition, respectively (Peslin, 1968). They were negative and positive, respectively, as expected in the presence of mechanical heterogeneity, recruitment/derecruitment, and gas trapping. These factors did in fact contribute to Δ*P*_alv_ more in elderly than in young healthy subjects, and markedly more in COPD patients (**Table 3**). This could be related to the presence of recruitment/derecruitment in patients in whom the closing volume exceeds the end-expiratory lung volume (Pecchiari et al., 2016). Indeed, if progressive airway collapse starts at volumes greater than the end-expiratory lung volume, the alveolar pressure inside the occluded regions should increase further with proceeding expiration, due to the reduction of the recoil of the surrounding non-occluded regions, thus elevating *P*_alv,ee_.

Several correlations were found between loop-derived parameters and spirometric and plethysmographic variables (**Table 4**), thus supporting the conclusion that loop-derived parameters reflect features of the ongoing pathophysiological processes in the lungs. For each loop-derived parameter, the strongest correlation occurred with inspiratory and expiratory airway resistance; this was expected, given the way *R*_ins_ and *R*_exp_ are computed, namely (*P*_mx,ins_-*P*_alv,ee_)/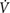
_(Pmx,ins)_ and (*P*_mx,exp_-*P*_alv,ei_)/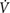
_(Pmx,exp)_, the pressure difference corresponding to the major axis of the inspiratory and expiratory loop, respectively.

The correlation between loop-derived parameters and routine lung function or exercise related variables was rather poor, and often not significant. The number of significant correlations, as well as the absolute value of the correlation coefficients were roughly similar for Δ*P*_alv_ and *A*_exp_, but substantially reduced for *A*_ins_, likely because of the smaller amplitude of the inspiratory loops (**Table 4**). However, there was a close relation between the corresponding correlation coefficients pertaining to Δ*P*_alv_ and *A*_exp_ or *A*_ins_ (*R* = 0.915 and 0.900, respectively), independent of whether these coefficients were significant or not (**Table 4**), indicating that all the loop-derived parameters had the same kind of relationship with the routine lung function or exercise related variables. Furthermore, the sign of the correlation coefficients of the relations between loop-derived parameters and routine lung function or exercise related variables were consistent with the expected modifications of all these variables with increasing disease severity. Quantification of loop-derived parameters could thus provide additional tools to better define the status of COPD patients.

### Limitations

This study suffers from several limitations.

Commercial plethysmographs may not be widely available, can be used only at rest and in the sitting position, and cannot be used under various physiologically interesting circumstances, like exercise testing, ICU settings, or at different postures. Moreover, measurements were performed with a particular commercially available plethysmograph (Jaeger Masterscreen Body Box), using an algorithm for thermohygrometric artifact compensation, the details of which have not been published, to our knowledge at least. A technical note (Peslin et al., 1996) has raised some doubts about the precision of this algorithm.

*P*_alv_–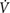 loops reflect the effects of factors operating during spontaneous tidal breathing at rest, while several variables reported in **Table 4** to which loop derived parameters have been related, were recorded during vital capacity maneuvers. For example, the amount of gas trapping as estimated by RV/TLC% or by a decrease of VC% does not ensure that gas trapping were present during spontaneous breathing.

Finally, in this study a quantitative relation between the magnitude of the loops or Δ*P*_alv_ and that of each factor involved in the formation of *P*_alv_ – 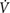 loops has not been established.

## Conclusion

The results of the present research show that the *P*_alv_–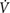 loops recorded in COPD patients during spontaneous breathing at rest contain information regarding the pathophysiological processes which characterize their lung mechanics, especially tidal expiratory flow-limitation and gas trapping. A complete identification of the factors responsible for the formation of the loop and their relative contributions was beyond the purpose of this observational study. The marked difference between the area of the expiratory part of the *P*_alv_–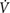 loop of elderly healthy subjects and COPD patients, the capability of tidal expiratory flow limitation to affect only this part of the loop, and the presence of tidal expiratory flow limitation in COPD patients but not in elderly healthy subjects suggest the possibility of a close relation between the magnitude of that area and tidal expiratory flow limitation, while the difference between end-inspiratory and end-expiratory alveolar pressure could provide a quantitative assessment of the impact of mechanical heterogeneity and gas trapping. Future investigations directed to this aim may extend the diagnostic capability of the plethysmographic technique, and provide an easy and cheap way to phenotype COPD patients.

## Author Contributions

DR, MP, and PS conceived the study. DR made the experiments. CZ, DR, ED, FP, and MP analyzed the data. CZ, DR, ED, MP, and PS drafted the manuscript. CZ, DR, ED, FP, MP, and PS critically revised the manuscript and gave final approval.

## Conflict of Interest Statement

The authors declare that the research was conducted in the absence of any commercial or financial relationships that could be construed as a potential conflict of interest.
